# The *FU *gene and its possible protein isoforms

**DOI:** 10.1186/1471-2164-5-49

**Published:** 2004-07-22

**Authors:** Torben Østerlund, David B Everman, Regina C Betz, Monica Mosca, Markus M Nöthen, Charles E Schwartz, Peter G Zaphiropoulos, Rune Toftgård

**Affiliations:** 1 Department of Biosciences at Novum, Karolinska Institutet, SE-141 57 Huddinge, Sweden; 2 J.C. Self Research Institute, Greenwood Genetic Center, Greenwood, South Carolina, USA; 3 Department of Medical Genetics, University of Antwerp, Antwerp, Belgium; 4 Institute of Human Genetics, University of Bonn, Bonn, Germany; 5 Pharmacia Corp., Milan, Italy; 6 Life & Brain Center, University of Bonn, Bonn, Germany

## Abstract

**Background:**

*FU *is the human homologue of the *Drosophila *gene *fused *whose product fused is a positive regulator of the transcription factor Cubitus interruptus (Ci). Thus, FU may act as a regulator of the human counterparts of Ci, the GLI transcription factors. Since Ci and GLI are targets of Hedgehog signaling in development and morphogenesis, it is expected that FU plays an important role in Sonic, Desert and/or Indian Hedgehog induced cellular signaling.

**Results:**

The *FU *gene was identified on chromosome 2q35 at 217.56 Mb and its exon-intron organization determined. The human developmental disorder Syndactyly type 1 (SD1) maps to this region on chromosome 2 and the *FU *coding region was sequenced using genomic DNA from an affected individual in a linked family. While no *FU *mutations were found, three single nucleotide polymorphisms were identified. The expression pattern of *FU *was thoroughly investigated and all examined tissues express *FU*. It is also clear that different tissues express transcripts of different sizes and some tissues express more than one transcript. By means of nested PCR of specific regions in RT/PCR generated cDNA, it was possible to verify two alternative splicing events. This also suggests the existence of at least two additional protein isoforms besides the FU protein that has previously been described. This long FU and a much shorter isoform were compared for the ability to regulate GLI1 and GLI2. None of the FU isoforms showed any effects on GLI1 induced transcription but the long form can enhance GLI2 activity. Apparently FU did not have any effect on SUFU induced inhibition of GLI.

**Conclusions:**

The *FU *gene and its genomic structure was identified. *FU *is a candidate gene for SD1, but we have not identified a pathogenic mutation in the *FU *coding region in a family with SD1. The sequence information and expression analyses show that transcripts of different sizes are expressed and subjected to alternative splicing. Thus, mRNAs may contain different 5'UTRs and encode different protein isoforms. Furthermore, FU is able to enhance the activity of GLI2 but not of GLI1, implicating FU in some aspects of Hedgehog signaling.

## Background

The signaling molecule Hedgehog (Hh) and components of its intracellular signaling pathway have been the subject of intensive research in several species from fruit fly to man during recent years. Numerous developmental and morphogenic processes are controlled by the Hedgehog family of proteins. Much effort has been directed at identifying components of the signaling pathway and their respective roles and interactions [for an extensive review see [1]]. In *Drosophila*, Hh signaling to the transcription factor Cubitus interruptus (Ci) is mediated by a protein complex consisting of Ci and three other cytosolic proteins. These are the costal 2 (cos2), suppressor of fused (su(fu)) and fused (fu), where fu is a kinase domain containing protein with positive regulatory activities in Hh induction of Ci mediated transcriptional activation. Hh binds to its receptor patched (ptc), a 12 membrane spanning protein, leading to the activation of another membrane protein smoothened (smo) [2,3]. Smo is a 7 transmembrane protein that, by an unknown mechanism, signals to the Ci containing protein complex leading to activation of Ci. Vertebrate homologues of these *Drosophila *genes and proteins have been identified during the last decade. To a large extent the signaling pathway has been conserved in vertebrates. However, the picture is more complicated since some of the *Drosophila *genes have two or more vertebrate homologues. There are three Ci homologues in vertebrates, GLI1, GLI2 and GLI3. GLI1 has activation properties whereas GLI2 and GLI3 have both activation and repression activities [reviewed in [4]]. It is expected that the human homologue of fu (FU) is a positive regulator of GLI proteins, whereas the su(fu) homologue SUFU is a negative regulator. It has been shown by several groups that SUFU inhibits both GLI1 and GLI2 transcriptional activity and has major effects on the shuttling between cytosol and nucleus [5-7]. In a similar way it was shown in C3H/10T½ cells that FU is a positive regulator of GLI2 but with little effect on GLI1 [8]. FU is a 1315 residue protein with high similarity to fu in the N-terminal kinase domain.

Interestingly, it was discovered that mutations in *PTCH1*, the human counterpart of *ptc*, underlie the Nevoid Basal Cell Carcinoma Syndrome (NBCCS) [9,10]. Patients with NBCCS (also known as Gorlin syndrome) have developmental abnormalities and eventually develop basal cell carcinoma (BCC) and other tumors like medulloblastoma and rhabdomyosarcoma [11,12]. Also *SMO *and *SUFU *mutations as well as overexpression of GLI1 or GLI2 can lead to BCC or medulloblastoma [13-16]. Thus, investigations of this signaling pathway, its genes and protein components, is not only important for understanding development and morphogenesis, but also for cancer biology.

Here three FU cDNA clones have been identified and used for sequence analysis, identification and structural description of the *FU *gene, as well as for construction and subcloning of FU expression vectors. Using the available public databases the *FU *gene was found to be present in a sequenced BAC clone from chromosome 2. *FU *is located in the same region of chromosome 2q34-q36 to which the human limb malformation disorder Syndactyly type 1 (SD1) has recently been mapped in a large German pedigree [17] and confirmed in an Iranian family [18]. Its possible association with this condition was investigated by sequencing the coding exons of the *FU *gene in an affected member from the German family [17]. The tissue expression pattern of *FU *has been determined using an RNA array and Northern blots. FU is expressed in all 72 tested tissues. It is clear that not only a single transcript is expressed. Instead transcripts of different sizes are seen and some tissues apparently express more than one major transcript. From the genomic structure and the cDNA clones it was possible to predict several alternative splicing events and consequently the likely expression of different protein isoforms. Two of the isoforms were expressed in HEK293 cells and tested for their ability to regulate the activity of GLI1 and GLI2, showing positive effects on GLI2 but not on GLI1.

## Results

### Chromosomal localization of FU

The sequence information derived from the FU cDNA clones 1HFU, 2HFU and Ngo3689 (see Methods) allowed the identification of the *FU *gene in a 200 kb BAC clone (AC009974) from chromosome 2. The gene is localized to 2q35 at 217.56 Mb using the Ensembl [19] annotation. The Ensembl gene prediction programs have identified most, but not all (21 of 29 exons; the published FU [8] predicts 26 exons) of the *FU *structure and named the gene STK36 (serine/threonine protein kinase 36). Chromosome 2q35 is the locus of several genetically based disorders. Both Syndactyly type 1 (SD1) and Brachydactyly type A1 (BDA1) have been mapped to this region [17,18,20]. Recently, the gene responsible for BDA1 has been identified as *IHH *(Indian Hedgehog) one of the vertebrate *Hh *homologues [21]. *IHH *is located in the vicinity of *FU *on chromosome 2 (217.94 Mb) less than 400 kb away. In order to determine if alterations of *FU *are responsible for SD1, the *FU *coding region (exons 3–29) and the flanking intronic regions were sequenced using genomic DNA from an affected member of an SD1 family whose trait maps to the 2q34-q36 region [17] and an unrelated control individual. No *FU *mutations were detected in this study, although three single nucleotide polymorphisms were identified. These included a T to C transition in intron 10, 17 bp 5' of exon 11 (IVS10-17T>C), causing gain of a BstNI site, and a G to A transition in exon 16, 17 bp 5' of the end of the exon (1748G>A), causing substitution of glutamine for arginine at amino acid 583 (R583Q) and loss of an AciI site. The altered restriction sites created by these sequence changes were tested in 44 CEPH unrelated individuals. The results showed that both changes are normal sequence variations as previously reported in the NCBI SNP database. The third change was a G to A transition in exon 27 (3008G>A), causing substitution of aspartic acid for glycine at amino acid 1003 (G1003D). By sequencing exon 27 in 8 affected and 6 unaffected members of the SD1 family [17], the disease variant could be observed in affected and unaffected members of the family, and a homozygous healthy individual was found. This variant has also been reported previously as a single nucleotide polymorphism in the NCBI SNP database.

### *FU *structure

**Figure 1:**
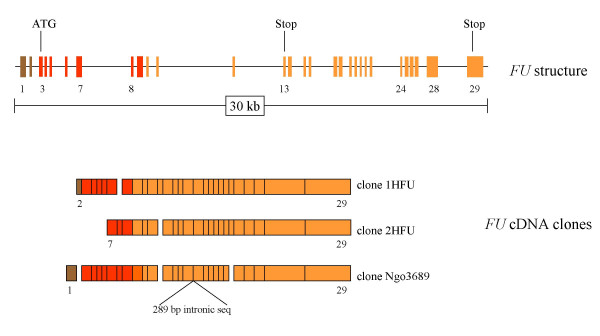
***FU *gene structure**. All sequenced segments of the available FU cDNA clones (1HFU, 2HFU and Ngo3689) were identified in a BAC clone (AC009974) from chromosome 2. This allowed the identification of the exonic sequences and thereby also the introns. The FU protein sequence [8] and translational analysis provided information about the role of different exons. Exons 1 and 2 encode 5'UTRs shown in brown. Exon 3 contains the initiating ATG codon approximately in the middle (position 90–92 from 5'end). Exons 3 to 9 encode the kinase domain shown in red. As judged from the cDNA clones the sequences encoded by exons 8, 13 and part of exon 24 are subjected to alternative splicing. Both exon 13 and 29 encode in frame stop codons. The cDNA clones end with a poly A^+ ^tail at the same position starting 706 bp from the stop codon (TGA) in exon 29.

None of the obtained cDNA clones contain sequence from all *FU *exons, but they allow determination of the exon-intron organization of *FU*. Figure 1 shows the structure of the *FU *gene. The cDNA clones are outlined to account for the predicted structure. Only clone 1HFU and Ngo3689 contain exons from the 5'non-coding region. To the 5' side of the sequence encoded by exon 3 these clones are different, indicating that alternative 5' untranslated regions (UTR) from different exons can be used. Exons 3 to 9 encode the N-terminal kinase domain. None of the cDNA clones encodes the FU protein that has previously been described [8]. 1HFU lacks the sequence encoded by exon 8, which results in a frame shift and a premature termination of translation. However, it cannot be unambiguously excluded that this may encode a very short protein isoform having only a partial kinase domain. 1HFU also includes the sequence from exon 13, which encodes an in frame stop codon. Neither Ngo3689 nor 2HFU contain the sequence encoded by exon 13. It is suggested that inclusion of exon 13 gives rise to a shorter protein (S-FU) of 474 residues, encode by exons 3 to 13. The previously described [8] long form of FU (L-FU) having 1315 residues is encoded by exons 3 to 29 without inclusion of exon 13. An additional alternative splice variant is suggested from the Ngo3689 clone. The first 63 bp of the sequence in exon 24 are missing. This results in a protein that is 21 residues shorter than L-FU (encoded by exons 3 to 29 without exon 13 and the 63 bp). Since almost all of the sequence from exon 24 is missing (only 18 bp are left) this isoform is termed L-FUΔ24. The Ngo3689 clone also contains all the 289 bp from intron 17, but whether this represents a true alternative splice variant is doubtful.

### Multi tissue array and northern blot analyses

**Figure 2:**
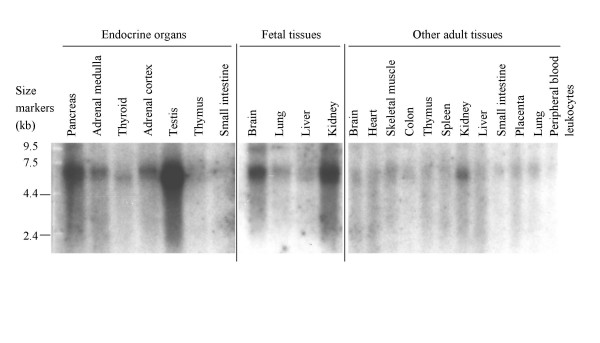
**Northern blot analysis of FU expression in human tissues**. Three commercially available Northern blots were hybridized with a labeled FU probe and analyzed by phosphorimaging. The blots have RNA from endocrine organs, other adult and fetal tissues as indicated. Size markers are shown to the left.

A tissue array with poly A^+ ^RNA from 72 human tissues was hybridized with a labeled probe 3' of the kinase domain. It was clear from this array that all examined tissues express *FU *to some extent. The highest amount of FU transcripts were detected in testis and pituitary (not shown). This is in agreement with the previous results by Northern blotting, showing highest *FU *expression in testis [8]. This analysis revealed that most tissues express an approximately 5 kb transcript [8]. The Northern blot analysis was here repeated with a larger number of tissues and a probe containing a 3' portion of the gene (exon 28). Figure 2 shows results of the three different Northern blots used. Here the transcripts are estimated to be a bit larger than the reported 5 kb, generally in the range of 6 to 7 kb. It should be emphasized that the identified cDNA clones are approximately 5 kb and that this seems closer to the correct sizes of transcripts, though they may appear larger on the Northern blots. Adult skeletal muscle, thymus, spleen, liver, small intestine, placenta, lung and leukocytes show a faint 6.5 kb band. However, the adult tissues brain, heart, colon and thyroid express a shorter transcript of 6 kb. Adrenal seems to preferentially express a band in the 6.5 to 7 kb range. In pancreas, fetal brain and fetal kidney it appears that at least two bands are expressed in the range from 6 to 7 kb. Besides, mRNA from fetal brain and lung also give rise to a band of much larger size around 9.5 kb. It is not clear if this constitutes a transcript that has not been fully processed, or whether it may contain sequences from as yet unidentified exons, for instance unknown 5'UTRs. Fetal lung and liver clearly preferentially express transcripts of different sizes, 7 and 6 kb respectively. It is confirmed that adult testis shows the highest expression but also pancreas, kidney, fetal brain and kidney stand out, in agreement with the previously reported Northern analysis [8]. Since one major alternative splicing event seems to involve exon 13, we attempted to evaluate the tissue specificity of this. A probe containing only the exon 13 sequence was used in hybridization of the Northern blots. This resulted in smeary bands irrespective of the hybridization conditions used (not shown). A likely explanation is that the probe is too short to achieve high specificity hybridization, or perhaps the transcripts containing exon 13 are degraded much faster, possibly due to the process of nonsense mediated decay [22].

### Nested PCR analyses

**Figure 3:**
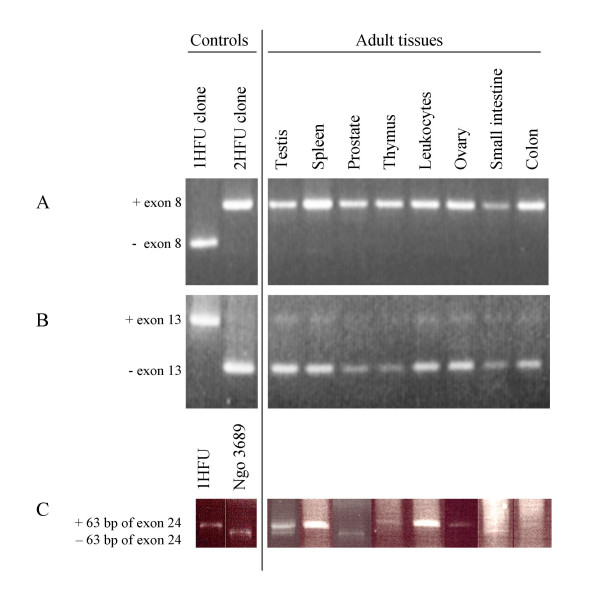
**Analyses of alternative splicing by nested PCR**. Nested PCR was performed on cDNA from 8 tissues, over regions implicated to undergo alternative splicing. These are encoded by exons 8 (panel A), 13 (panel B) and 24 (panel C). Available cDNA clones either with or without these parts served as controls as indicated. The PCR products were analyzed on agarose gels as shown.

To examine more specifically the expression of exons that are involved in alternative splicing events, nested PCR was performed on cDNA probes generated from whole tissue RNA by reverse transcription. Sequences containing segments including exons 8, 13 and 24 were amplified and analyzed on agarose gels (Fig. 3). It was not possible to detect transcripts that lack exon 8 (Fig. 3, panel A). In contrast, it appears that transcripts both with and without exon 13 are present (Fig. 3, panel B). Expression of transcripts without exon 13 is clearly most prevalent in all tissues. The expression of the longer form seems to be proportional to the expression of the shorter form. This indicates that this splicing event is not subjected to any significant tissue specific regulation. However, since a detectable amount of transcripts including exon 13 are present, it is likely that S-FU is also expressed in the tissues. The analysis of alternative splicing of the part encoded by exon 24 turned out to be difficult and several primer pairs were tested before reliable results could be obtained (Fig. 3, panel C). Interestingly, the examined tissues show very different expression patterns, suggesting that alternative splicing in this case is a regulated event. Most tissues express all of exon 24 but in small intestine and prostate clear expression of a transcript without the 63 bp is observed, and in testis expression of transcripts both with and without this segment is found. Sequence analysis shows that the 63 bp segment is likely to encode part of a leucine zipper domain and therefore a putative protein interaction may be lost in this isoform (L-FUΔ24).

### Functional analyses of FU isoforms

**Figure 4:**
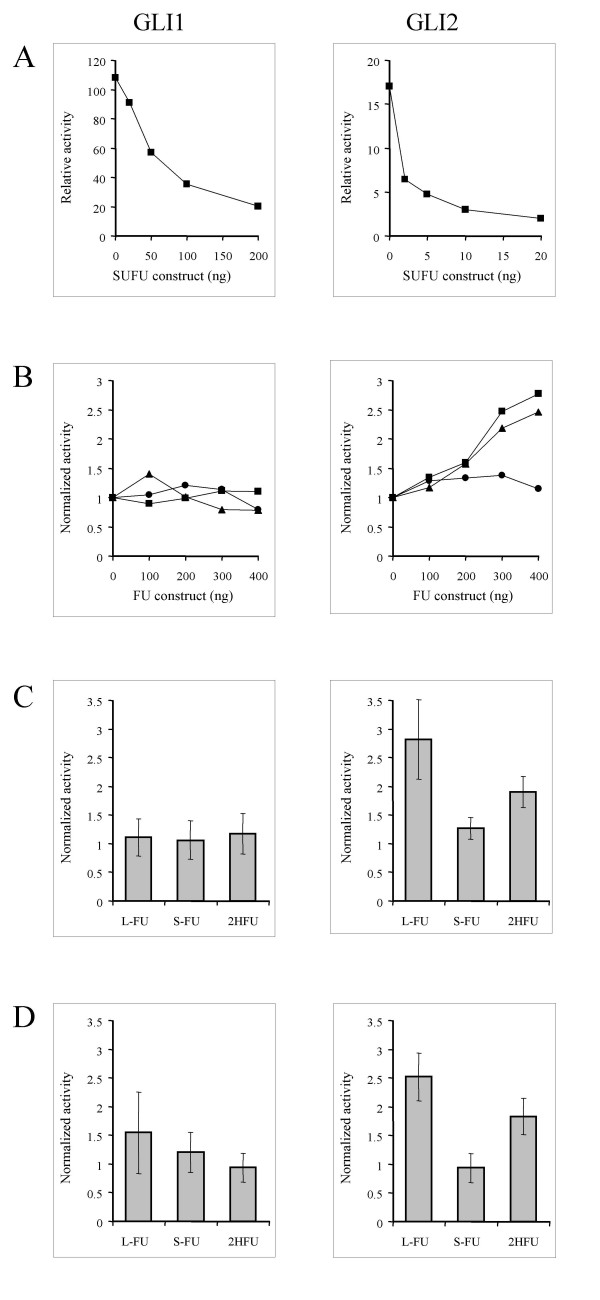
**Regulation of GLI induced transcriptional activity in transfected HEK293 cells**. HEK293 cells were transfected with a GLI inducible luciferase reporter construct together with a GLI1 or GLI2 expression construct. The cells were also transfected with a β-galactosidase construct that served to correct for transfection efficiency and cell density. The effects of FU and SUFU were tested by cotransfecting FU and SUFU expression constructs and compared to effects with empty vectors. Panel A shows typical examples of SUFU effects on GLI1 and GLI2. Panel B shows typical examples of the effects of different FU constructs on GLI1 and GLI2; experiments that were performed in parallel are shown, to illustrate the different impact on the GLI proteins. L-FU is shown with squares, 2HFU with triangles and S-FU with circles. Panel C shows the impact of FU constructs (400 ng) on GLI1 and GLI2 as summarized by the results of at least 4 different experiments. Panel D shows the impact of FU constructs (400 ng) on SUFU inhibited GLI1 and GLI2 as summarized by the results of 3 different experiments. In these experiments the GLI induced transcriptional activity was inhibited to 20–40 % of the non-inhibited level by the addition of SUFU (i.e. 2½ to 5 fold inhibition). Relative activity (panel A) is given as compared to activity in mock transfected cells and normalized activity (panel B-D) is given relative to activity in cells transfected with GLI and GLI+SUFU set to 1.

Investigations into the ability of FU isoforms to regulate GLI transcription factors and SUFU has here been initiated by expression of both L-FU and S-FU as well as 2HFU, which does not have a full kinase domain, in HEK293 cells. The 293 cells, unlike the previously used C3H/10T½ cells [8], do not have a complete Hedgehog signaling pathway. Thus, it is possible to determine if FUs have direct effects on GLI proteins as it was done for SUFU [5]. The assay is based on the induction of a luciferase reporter construct having 12 consecutive binding elements for GLI transcription factors [5]. In all transfection assays GLI1 was able to induce the luciferase reporter 100–250 fold, whereas GLI2 induced the reporter some 15–30 fold, depending on cell density and the amount of construct used. Figure 4 shows the results of these expression analyses. As shown previously [5] SUFU has a strong inhibitory effect on GLI1. Moreover, a similar strong effect was seen on GLI2 (Fig 4., panel A). The results presented are typical for a large number of experiments and depend on the amount of GLI and SUFU that is used. In contrast to the strong effects seen with SUFU, none of the FUs revealed major changes (Fig. 4, panel B and C). It is clear that the FUs are not able to regulate GLI1 at all, though L-FU and 2HFU have a weak (2–3 fold) positive effect on GLI2. It appears that L-FU has a slightly stronger effect than 2HFU. This is qualitatively the same result as was obtained in C3H/10T½ cells, where the L-FU was also compared to a kinase-dead mutant and a 546 residue variant similar to S-FU. Thus, in both cases there is no evidence that the kinase domain is required for the activation of GLI2. Unlike the previous analyses [8], it cannot be confirmed that FUs have a direct effect on SUFU function (Fig. 4, panel D). The inhibitory effect of SUFU on GLI1 is not relieved by the addition of FU. The effect on GLI2/SUFU cannot be distinguished from the effect on GLI2 alone, implying that FU does not regulate SUFU but may only affect GLI2. Since 293 cells lack components of the Hedgehog signaling pathway, it is possible that an effect of L-FU on SUFU [8] requires the presence and activity of additional molecules.

## Discussion

### The *FU *gene

In the present paper, the *FU *gene was identified and its structure determined. *FU *consists of 29 exons of which exons 1 and 2 encode 5'UTRs, exon 3 contains the initiating ATG codon and exons 13 and 29 contain in frame stop codons (Fig. 1). Exon 1 and 2 may serve as alternative first exons, like the alternative exons 1, 1A and 1B found in the *PTCH1 *gene [23]. Exons 3 to 9 encode the kinase domain. This segment has strong similarity to Drosophila fu, whereas the remaining C-terminal part has a much weaker similarity [8]. Using the DIALIGN program [24] it is possible to align fu to L-FU in two regions in the C-terminal part (not shown). These are largely encoded by exons 15–16 and 22–29. This indicates that exons 10–14 and 17–21 may have been recruited to the *FU *gene during evolution.

### Investigations of Syndactyly patient material

We investigated the possibility that *FU *underlies SD1 based upon the fact that *FU *lies within the localization interval for SD1 and that it is part of the Sonic Hedgehog signaling pathway, which participates in digital patterning [1]. Although three previously reported single nucleotide polymorphisms were identified, we did not detect any mutation in the *FU *coding region or flanking intronic regions. While these results do not implicate *FU *in the causation of SD1, it is possible that this disorder is caused by mutations in the noncoding regions not screened in this study. Alternatively, SD1 could be caused by a genomic rearrangement not identified by sequence analysis, although no altered bands were detected in an affected member of the SD1 family by Southern analysis using a *FU *cDNA clone as probe (data not shown).

### Expression analyses

Analyses of *FU *expression have shown that transcripts are detected in all tissues examined. For the first time evidence is presented showing that more than one transcript can be expressed from this gene. The Northern blots clearly show that FU transcripts of different sizes indeed exist. Here the transcripts are estimated to be slightly bigger than previously reported and in some tissues more than one transcript is evident. It is clear from the available cDNAs and RT/PCR based transcript analyses that alternative splicing occurs. Additionally, it is also clear that different 5'UTRs are present in the transcripts. At least two protein isoforms, besides the previously described L-FU [8], may be produced. The S-FU isoform is the one that most dramatically differs from L-FU, consisting only of the N-terminal one third of L-FU. S-FU expression results from inclusion of exon 13 in the mature transcript. This alternative splicing event was detected in all tissues examined and at an apparently constant ratio. Also a case of regulated alternative splicing was detected by RT/PCR, but with a much less dramatic impact at the protein level, since it only results in the loss of 21 residues encoded by exon 24. However, the expression reveals a possible tissue specific regulation of this alternative splicing event. This may well reflect that L-FUΔ24 plays a biological role different from L-FU. Since it appears that the mRNA for L-FUΔ24 is not expressed in small intestine and prostate it can be speculated that FU has a different role there, if a leucine zipper is truly lost in L-FUΔ24. It is intriguing that testis appears to express transcripts both with and without the 63 bp segment and is also the tissue with strongest expression. Perhaps the expression of L-FUΔ24 and L-FU together is linked to the function of Desert Hedgehog which has been shown to have a particular role in spermatogenesis [25]. Whether interactions with GLI proteins, SUFU or other components of the signaling pathway are altered, and if this has any impact on GLI or SUFU activities, remains to be investigated. Certainly this adds another variable to the complicated picture of Hedgehog signaling and GLI regulation in vertebrates.

### Functional investigations and perspectives

The assessment of functionality revealed that S-FU was not able to regulate GLI1 or GLI2 when expressed in 293 cells. In contrast, both L-FU and a variant lacking a full kinase domain (2HFU) were able to enhance GLI2 induced transcription. These results are qualitatively similar to those previously reported in C3H/10T½ cells [8]. L-FU and 2HFU were only able to enhance GLI2 activity 2 to 3 fold in 293 cells, whereas 5 to 8 fold inductions are seen in C3H/10T½ cells. This may reflect the fact that the latter cell line expresses additional components of the Hedgehog signaling pathway, which are required for full activity of FU. Unlike the previous investigations [8] it was not possible to see an effect of L-FU on SUFU. Again this difference may be explained by the various properties of the cell lines used. Understanding the signaling events downstream of SMO may reveal functional differences of the proteins involved, as compared to their fruit fly counterparts. Although SUFU inhibits GLI transcription factors and su(fu) inhibits Ci, there are still striking differences. As yet there have been no reports of a cos2 counterpart in vertebrates. Instead it has been observed that FU interacts with all GLI proteins and SUFU [8], even though fu does not bind to Ci [26]. It has also been observed that both L-FU and SUFU can be found in the nucleus [5-8], which has not been observed for fu or su(fu). It is likely that both FU and SUFU are shuttled in and out of the nucleus by binding to GLI proteins [5,8]. Though basic activities of both FU and SUFU in regulation of GLI have been conserved, it also appears that significant differences from their fruit fly counterparts exist. Clearly, FU is not having an effect on GLI1 similar to the one seen on GLI2. Additional investigations are needed in order to establish the role of FU in hedgehog signaling and GLI control. The role of the different isoforms also remains to be elucidated. These have to be tested individually for their regulation of all GLI proteins and proteolytic products. Fu is known to have at least two separate physiological functions in the fly, one of which is dependent upon the kinase domain [27]. Likewise, FU may well have two or more distinct functions in signaling, represented by different domains, isoforms and protein interactions.

## Conclusions

*FU *is localized on chromosome 2q35 very close to *IHH*. Though SD1 has been mapped to this region, we have not identified a causative role for *FU *in this disorder. *FU *consists of 29 exons of which 1 and 2 encode 5'UTRs and 3 to 9 encode a kinase domain. For the first time it is shown that transcripts of different sizes are expressed and alternative splicing takes place, probably leading to the generation of different protein isoforms. FU protein is likely to be involved in the Hedgehog signaling pathway since it can enhance the activity of GLI2. In contrast, it has no effect on GLI1 and an effect on SUFU cannot be observed in 293 cells.

## Methods

### The FU cDNA clones

Two almost full-length human FU clones were identified in the Incyte database. Both 1HFU and 2HFU were cloned in the vector pINCY. A third clone was available from Kazusa DNA Research Institute (Chiba, Japan) and termed Ngo3689 (Gene name KIAA1278). This clone was in the vector pBluescript II SK^+^. The human BAC clone AC009974 was obtained from Research Genetics (Huntsville, AL). The human GLI, human SUFU, 12GLI-RE-luciferase reporter and β-galactosidase vectors have been described previously [5].

### FU cDNA subcloning

Expression constructs for different isoforms of FU was obtained by direct PCR or extension overlap PCR, using end-primers having specific restriction sites and the high fidelity Vent_R _DNA polymerase (New England Biolabs, Beverly, MA). The cDNA for the long form of FU (L-FU) was subcloned into pCDNA3.1-HisB using the NotI and XbaI sites. 2HFU and the short FU (S-FU) cDNAs were subcloned into pCDNA3.1-HisC using the KpnI and XbaI sites.

### DNA sequencing and analyses

All PCR generated products were analyzed by DNA sequencing. The Big-Dye Terminator Cycle Sequencing kit (Applied Biosystems, Foster City, CA) was used according to instructions. Sequencing was performed at CyberGene AB (Huddinge, Sweden). Sequence alignments were done using the DIALIGN program [24] available at the BiBiServ from University of Bielefeld, Germany. Sequence information of proteins, clones and chromosomes were obtained from the Swiss-Pro [28], Entrez [29] and Ensembl [19] databases.

### Analyses of genomic DNA from family members with SD1

After informed consent was obtained, blood was taken from affected and unaffected family members and DNA extracted from peripheral blood leukocytes according to standard methods. Intronic primers were designed to amplify exons 3–29 of *FU *either as single exons with flanking intronic sequences or as products containing two exons with flanking intronic sequence and the complete intervening intron. The primer sequences can be obtained upon request. PCR was performed in a standard fashion and products were sequenced using either the Thermosequenase CyTM5.5 Dye Terminator or DYEnamic ET Dye Terminator Cycle Sequencing kits (Amersham Biosciences, Piscataway, NJ). Electrophoresis and analysis were performed on either an Automated Laser Fluorescence (ALF) DNA sequencer or MegaBACE DNA sequencer (Amersham Biosciences) after purification with Autoseq columns (Amersham Biosciences). For exon 27, the PCR product was purified using the enzymatic ExoI-SAP purification method, sequenced using the Terminator Cycle Sequencing kit (Amersham Pharmacia Biotech) and analysed on an ABI 3100 genetic analyzer (Applied Biosystems). PCR products containing exon 11 or exons 15/16 were digested with BstNI or AciI, respectively, and the bands resolved on 3–4% agarose gels to confirm sequence changes in the patient with SD1 and to determine their frequency in a panel of 44 CEPH individuals.

### Northern blot analysis

Commercially available Human MTN 12-lane Blot 2, Human Fetal MTN Blot II and Human Endocrine System MTN Blot Northern blots (Clontech, Paolo Alto, CA) were obtained and used with PCR generated hybridization probes. DNA probes were made by direct PCR, amplifying the sequences corresponding to exon 13 and 28. The generated fragments were then labeled with ^32^P-ATP using the High Prime DNA labeling kit (Boehringer Mannheim, Mannheim, Germany) according to instructions. Hybridization of Northern blots was done with labeled DNA probes in ExpressHyp (Clontech) at 68°C according to instructions. The blots were then analyzed with a Fujix Bas 2000 phosphoimager (Fuji Photo Film, Tokyo, Japan).

### Expression analysis by nested PCR

The expression of exon 8, 13 and 24 sequences in mRNA was assessed by nested PCR on RT/PCR generated cDNA samples from eight different tissues as provided in Human Multi Tissue cDNA Panel II (Clontech). Two sets of primers were made for each exon to be investigated. The outer pairs were used in a first PCR using 5 μl of the cDNA and Vent polymerase. In a second PCR 0.5 μl of the first PCR products was used together with the inner primer pairs. These pairs were also used for PCR of FU cDNA clones that served as controls. The primer pairs are listed in Table 1. The PCR reactions were performed using 95°C for 1 min denaturation, elongation at 72°C and 40 cycles. The exon 8 and 13 sequence PCRs were performed using 60°C 1 min annealing and 1 min elongation. The exon 24 sequence PCRs were performed using 59°C 1 min annealing and 1 min 30 sec elongation.

**Table 1:** Primers for nested PCR analyses

Sequence from exon		Outer PCR primer pairs	Inner PCR primer pairs
8	fwd	5'-AACATCCTCCTCGCCAAGGGT	5'-ATATGAACTGGCAGTAGGCAC
	rev	5'-TGCTCTCCTGACTGT GCCTGAGTAGACTCA	5'-TTACCCTTGGGGGCCAACCGA
13	fwd	5'-AACATCCTCCTCGCCAAGGGT	5'-AGCCTGTGCCTATTCAACTGA
	rev	5'-TGCTCTCCTGACTGT GCCTGAGTAGACTCA	5'-GCCTCCCGGCAGAAGGAATAC
24	fwd	5'-CGCAAGTGAGCCAGCCACTGC	5'-CAGCCAGCTCAGGCCATCCCT
	rev	5'-CTGGACCGCAGGAATCT GGAATCACATGCTATGGG	5'-CCAGGCCTGTGAGAAGGCTGA

### Reporter gene assays

The cDNA clones were used in transfections of HEK293 cells in 24 well culture plates. Basically this was done as previously described [5]. In short, the 293 cells were transfected using Superfect Transfection Reagent (Clontech), with 100 ng of the luciferase reporter and β-galactosidase as well as different amounts and combinations of GLI, FU and SUFU constructs. For every assay there was a corresponding control with an equal amount of empty vector. The cells were harvested 24 hours after transfection with 50 μl of lysis buffer from the Galacto-Light kit (Applied Biosystems). Of this was 10 μl used for β-galactosidase assay and the rest for luciferase assay using the Luciferase Assay kit (BioThema, Dalarö, Sweden). Analyses were done on a Microplate Luminometer (Berthold Detection System, Pforzheim, Germany).

## Authors' contributions

TØ contributed to the experimental design; participated in sequencing, sequence analysis and subcloning; did the gene analysis, Northern blots, nested PCR, cell experiments; and made the manuscript draft. DBE and CES designed and carried out the patient analysis; and contributed to the manuscript. MM provided clones; contributed with subcloning; made the array analysis; and contributed to the manuscript. MMN and RCB provided the SD1 patient material; performed segregation analyses in the SD1 family; and edited the manuscript. PGZ contributed to the experimental design and subcloning; and edited the manuscript. RT contributed to the experimental design; did data base analysis; and edited the manuscript.
